# Serendipity in Relationship: A Tentative Theory of the Cognitive Process of *Yuanfen* and Its Psychological Constructs in Chinese Cultural Societies

**DOI:** 10.3389/fpsyg.2016.00282

**Published:** 2016-03-01

**Authors:** Hsin-Ping Hsu, Kwang-Kuo Hwang

**Affiliations:** Psychology, National Taiwan UniversityTaipei, Taiwan

**Keywords:** fate, *guan-xi*, serendipity in relationship, social and personal relationship, *yuanfen*

## Abstract

The main purpose of this article is to combine three important themes in Chinese cultural societies: serendipity in relationship (yuanfen), relational interactions, and psychological adaptation through self-cultivation. People who live in Chinese cultural societies are deeply affected by relationalism and tend to be very different from their Western counterparts, who adopt individualistic methods when dealing with interpersonal problems. They are highly likely to access the perspective of yuanfen as part of their cultural wisdom to convert negative feelings, awkwardness, or setbacks caused by interpersonal relationship incidents, into a type of cognitive belief that can be used to combat anxiety and actuate coping actions. Based on this, this article proposes the tentative theory of a dialectical model which comprises elements of the philosophies of Daoism, Buddhism and Confucianism, to analyze the cognitive operation process regarding yuanfen and to explain and predict how people in Chinese cultural societies differ from most Western people in terms of psychological adjustment and coping actions when dealing with interpersonal problems. Canonical correlation analysis was used in the empirical study to describe this model and resulted in two statistically significant canonical factor pairs. The hypothesized model has been partially verified. It is hoped that this framework can serve as a pilot perspective for future studies, and at the same time provide the Western academic world with a reference for understanding the concept and substantive effects of serendipity in relationship. Further suggestions for future research direction are offered.

“Relationalism” is a fundamental premise for studying interpersonal relationships in Chinese cultural societies. The studies based on relationalism will supplement the inadequacies of social psychological theory and research outcomes that have long been dominated by the Western perspective of “individualism,” particularly in the issue of interpersonal relationships. People who live in Chinese cultural societies are deeply affected by relationalism, and often apply different rules of engagement that change depending on their relationships with people around them. Compared to the people living in individualistic societies, it is more likely for them to experience intense psychological conflict between intrinsic drives and social norms when encountering interpersonal dilemmas. Therefore, researchers should focus on the psychological integration of individuals in various specific social contexts, and explore how people find a balance between the individual at the biological level and the ideal person at the social level (Hwang, 2011). From the perspective of Chinese psychology, this process of seeking intrinsic self-integration and pursuit of psychological equilibrium can be referred to as “self-cultivation.” This is a unique cultural practice that may motivate an individual to take the most suitable and ideal action under specific circumstances (Hwang, 2012), especially when people encounter interpersonal difficulties. In order to complete “self-cultivation,” they often seek solutions from the cultural wisdom accumulated in their existing cognitive schemas to restore “psychosocial homeostasis” (Shang and You, 2010; Hwang, 2011). As theorized by Francic L. K., Hsu, a psychological anthropologist, “psychosocial homeostasis” is the state of good balance between an individual and his social world, in particular when dealing with various interpersonal problems (Hsu, 1971, 1985).

Among various types of cultural wisdom, *yuanfen* (serendipity in relationship) and *mingyun* (fate) are practical methods of attribution, and dialectical beliefs commonly used by people who live in Chinese cultural societies, to interpret one's statuses of social interactions (Chang and Holt, 1991; Yeh, 2002; Yang, 2005). *Yuanfen* is an indispensable “serendipity” in relationship which implies that “chance” or “destiny” is hidden under any relationship; even when the relationship comes to an end. It is an interpersonal wisdom for flexible decision-making and psychological adaptation in Chinese cultural societies. In Chinese language, the words *yuanfen* is composed of two meanings from two characters—one representing fatalistic (*yuan*) and the other, voluntaristic (*fen*). Similarly, *mingyun* (fate) is also composed of two characters—the first one represents destiny (*ming*) and the other, fortune (*yun*). These Chinese cultural beliefs form a pair of contrasting opposites, which lead to specific beneficial results when dealing with various interpersonal problems. However, the word, “fate,” usually induced negative association for people who live in individualistic cultural societies. In fact, it is not really passive or negative in Chinese cultural societies; it may serve as the foundation of *yuanfen* and may be the key reason to make *yuanfen* work.

As an attributional thinking and defense mechanism used to solve interpersonal problems in Chinese cultural societies, *yuanfen* is not only a guiding directive of self-interpretation, but also widely applied in daily life. For example, any relationships must be based on *yuanfen* which is also the reason why these relationships occur or come to an end. A good and long relationship, such as a happy marriage can be called *liangyuan* while an unsuccessful and doomed long relationship can be referred to as *nieyuan*. Similarly, married couples who have a good relationship with one another indicates that they get heavenly blessings and can be considered as *ming hao*. On the other hand, married couples who have a bad relationship with each other implies that they do not have heavenly blessings and can be regarded as *ming bu hao*. Therefore, in Chinese cultural societies, when an individual is faced with interpersonal problems, these two concepts can motivate the individual to perceive “self-cultivation” as the means for getting through difficulties. If appropriately utilized and applied, these concepts may resolve the stress caused by the relational interactions, and even affect relationship satisfaction (Chang and Jou, 2004; Lee, 2005). Besides, some researches in Taiwan showed that the belief in *yuanfen* is significantly correlated with mental health and well-being (Lee and Chen, 2006, 2009). Hence, we hold that *yuanfen* can be drawn on in interpersonal situations to take the ideal coping actions in Chinese cultural societies. These ideal actions are expressed through expectations for “self-cultivation,” such as: forbearance, forgiveness, effort-making, and gratitude. Through these actions, one may satisfy the personal need for self-esteem and meet social value expectations, in turn achieving a psychosocial homeostasis.

Based on the description above, this paper proposes the tentative theory of a dialectical model of *yuanfen* (Figure 1), in an attempt to explain the cognitive operation process of psychological adjustment of people who live in Chinese cultural societies when dealing with interpersonal problems.

**Figure 1:**
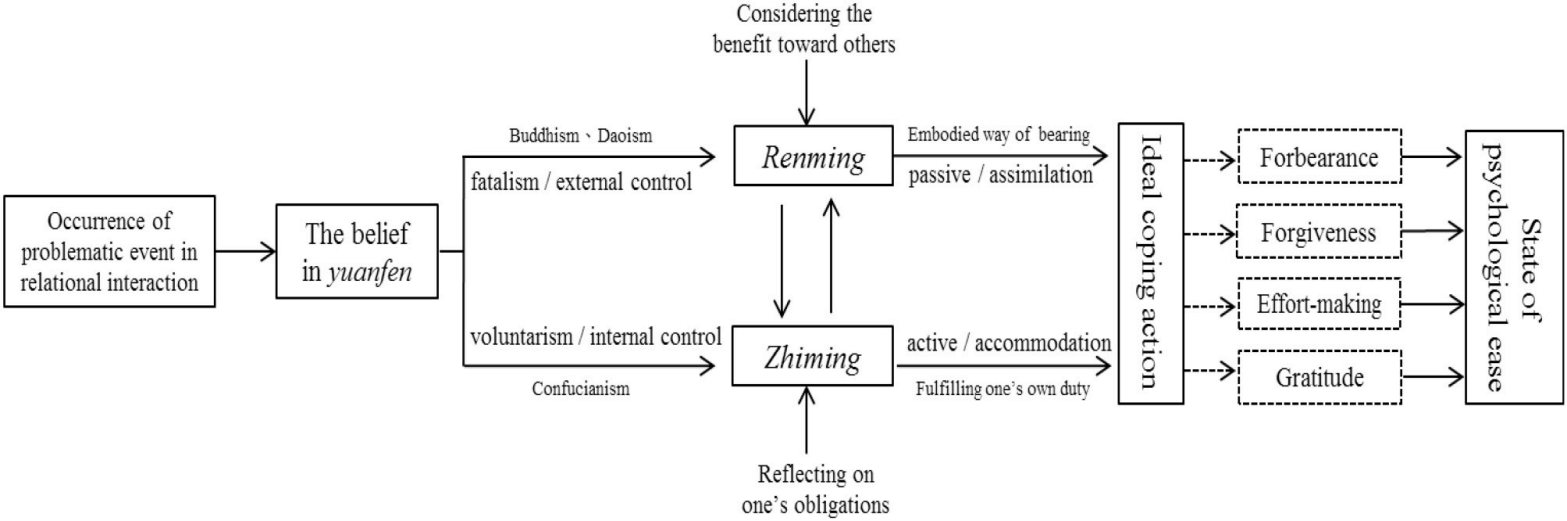
**A dialectical model of *yuanfen* and its function on psychological adaptation**.

## Culture inclusive psychology: the perspective in social and personal relationship study in chinese cultural societies

During a person's lifetime, cultural traditions may operate psychologically through heuristic processing. Accumulated through time and life experiences, these cultural traditions gradually become thoughts or habits that can be used to handle problems by the majority of the people in a society, which forms a cultural mentality unique in comparison to other societies. Such cultural mentalities affect how people adapt to their lives, and can be used as a method for self-healing. Since Chinese cultural societies are affected by relationalism, people tend to be very different from their Western counterparts, who take on individualistic ways in dealing with interpersonal problems.

According to Hwang (2011), if a person can use the habitus (Bourdieu, 1990) of normal action to smoothly handle life events under certain social conditions, it is unlikely that they will engage in deep reflection. However, if habitus cannot be used to resolve a problem, the person will attempt to seek solutions from their personal stock of knowledge or social stock of knowledge. The former include schema, as proposed by Piaget (1977), while the latter are cultural traditions (Shils, 1981). In other words, some cultural traditions are instrumental to problem-solving, and provide the crucial origins for the creation of cognitive schemas. When a person encounters difficulties and a certain method from social stock of knowledge is found to be effective, it may be incorporated into one's personal stock of knowledge for future application.

In Chinese cultural societies of relationalism, the psychological stresses elicited by interpersonal incidents tend to arise from significant others. For instance, the marital tensions between a couple may not necessarily be caused by themselves, but due to the involvement of their natal families. Therefore, in dealing with interpersonal issues, one cannot overlook significant others and situational contexts. Based on their life experiences, people are accustomed to appeal to *yuanfen* to convert negative feelings, awkwardness, or setbacks caused by interpersonal incidents, into a type of belief that can be used to combat anxiety. Its true functional mechanism is in embodying the perspective of the mandate of Heaven (Wang, 1987; Lee, 1995; Yang, 2005; Hsu and Hwang, 2013). These beliefs become practical wisdom or mechanisms of psychological adaptation for handling interpersonal problems. People use *yuanfen* to interpret the problem, and in turn adopt suitable actions to achieve psychological adjustment. *Yuanfen* demonstrates that people who live in Chinese cultural societies are accustomed to taking a continuous rather than fragmented perspective toward various interpersonal issues. They believe that the formation and destruction of various relationships may connect the past, present, and future as causes and consequences on the same timeline. This is particularly true for expressive ties that satisfy personal, intrinsic needs for love, warmth, security, and sense of belonging, such as parent-child, romantic, marital, and intimate relationships (Hwang, 2012), and may produce different judgments based on whether such expressive ties are inherent or learned.

In the field of Eastern psychology, *guan-xi*, a similar concept but not the same as “relationship” in Western psychology, has long been an important issue. However, existing literature has tended to focus on the explicit “*guan-xi* as it ought to be” rather than on the implicit “*guan-xi* as it is.” According to Zhai (1993), in Chinese society, there are three localized concepts for interpersonal relationships: personal appeal (*ren yuan*), human sentiment (*renqing*), and human relations (*renlun*). These three concepts correspond, respectively, to psychology, values, and norms, in turn creating an overall framework for the exploration of interpersonal relationships. This study postulates that human sentiment and human relations correspond to the explicit “*guan-xi* as it ought to be,” which can satisfy the expectations of Chinese social values and norms, but are also the sources of psychological disturbances. Since personal appeal corresponds to psychology, and is related to the overall configuration of the model of interpersonal relationships, it should have the most direct impact on psychological adaptation as part of relational interaction. For example, when a person forced to accept a breakup and attribute the failure of the relationship to lack of *yuanfen*, the relationship has also been framed as something that does not have to be taken seriously. Since there is a lack of *yuanfen*, the relationship should not be fought for. This interpretation is actually beneficial for psychological adjustment in terms of achieving a positive outcome.

Therefore, this paper postulates that adaptation in terms of interpersonal relationships should involve be a balance between personal appeal, human sentiment, and human relations in Chinese cultural societies. If the views of Harris (1989) and Hwang (2011) are incorporated, then human sentiment and human relations may be classified in the social culture dimension. In order to meet certain value expectations imbued by society, such as being an ideal person, social values and norms need to be taken into consideration in order to achieve a state of integrated psychological equilibrium. Considering the “Mandala Model of Self” postulated by Hwang (2011), at this point a person encountering difficulties would experience an internal tug-of-war, and would attempt to integrate primitive motivating drives and social norm requirements, the ideal action after reflection can be used to achieve the adjustment goal. This psychological process of seeking intrinsic self-integration can be referred to as self-cultivation, which has become a unique healing model in the Chinese culture. It stresses the situational context of problems, and can be used as an important basis for the construction of a localized psychological treatment model (Chen, 2009; Hwang and Chang, 2009; Leung and Chen, 2009; Hwang, 2012). Complemented with the views of “investigation of things, extension of knowledge, sincerity of the will, rectification of the mind, cultivation of the personal life” in *Great Learning* of Confucianism^1^, the cultivation process is the work of the self. People use their own wisdom and knowledge to achieve an understanding of the dilemmas they face, as a result, they complete themselves and complete others, and ultimately achieving a state of ease with adaptation between subjective and objective conditions (Hu, 2009). At this time, personal appeal, human sentiment, and human relations can achieve a stately good balance. This not only helps one to understand the various problems encountered in relational interactions, but also to integrate the three major systems of psychology, values, and norms, so that the self can realize its greatest potential.

## Buddhist, daoist, and confucian philosophies and the dialectical thinking process in the perspective of *yuanfen*

*Yuanfen* is frequently mistaken to be a purely Buddhist concept, but its content actually integrates Confucian, Daoist, and Buddhist wisdom. Because *yuanfen* has the Chinese conceptual element of unification of Heaven and human, which is an important belief advocated by Confucianism and Daoism. Therefore, people are accustomed to considering interpersonal relationships as part of their relationship with Heaven, creating the cultural knowledge of oneness of heaven, earth, and human sentiment and relationships (Zhai, 1993). Thus, “Heaven” may be a synonym for “nature,” symbolizing a state that occurs without deliberate intervention. In the human world full of dilemmas, it is a concept of transcendence, generally referring to the original nature of all objects (Huang, 2006). Therefore, comprehension of the meaning and evolution of *yuanfen* requires an understanding of Confucian, Daoist, and Buddhist views on interpersonal relationships.

Confucianism places greatest emphasis on ethics in interpersonal relationships, so it stresses the obligation to shoulder one's responsibility; Daoism places emphasis on naturalness in interpersonal relationships, so it stresses the ease of going with the flow; and Buddhism places emphasis on cause and consequences in interpersonal relationships, so it stresses the mercy of letting go. These three philosophies of life tend to appear through mutually dialectical conversion in the processing of interpersonal problems in Chinese cultural societies, becoming the sources of content for the cultural belief of *yuanfen*. Since troubles and suffering are only natural, and may happen again at any time, if one is desperate to dispel suffering from one's consciousness, this would only be the temporary suppression of a problem and one would not be able to achieve true liberation. Therefore, the ultimate expression of cultivation in Chinese cultural societies is the psychological transition from learning how to adjust to problems to converting them into positive interpretations. This also allows for the preservation of “face” (Hwang and Chang, 2009) and achieves a form similar to “self-verbalization” in Western theory. This kind of interpretation and application has affected Dialectical Behavioral Therapy (DBT) in the West, which has been proven to be effectively applied to the clinical treatment of patients with unstable interpersonal relationships (Linehan et al., 2006). However, self-verbalization that can truly benefit complicated interpersonal relationships must be the “self-dialectic” that can simultaneously combine thought from Buddhism, Daoism, and Confucianism to be the most effective practice.

Furthermore, Daoism stresses the following of the natural will of Heaven, so when people encounter problems, it would be best to maintain stillness and avoid actions without thinking, because heavenly principles would arrange fate. This view corresponds to the cause and consequences stressed by Buddhism. As the Buddhist aphorism states, “Nothing will follow one in death except for karma.^2^” If one wants to be freed from pain and suffering, one must maintain a merciful heart. This perspective would make it easier for one to see random encounters or interpersonal problems as fated to come because of causes and consequences, as a natural rhythm or flow. If one can freely accept difficult challenges, one would be able to implement embodied way to cultivate and elevate one's psychological state. Because one can account for the limitations of the objective relationship context, believing that any relationship involves necessary causes, problems and troubles are merely consequences and outcomes. Therefore, under certain interpersonal contexts, one would choose to forbear in response. Moreover, the consideration of another person's own karma would better enable one to exercise mercy and empathy, and as a result, people can forgive and liberate one another. Understanding this point allows one to achieve happiness, changing one's perception of the dilemmas originally present in the relationship. Thus, under the profound concept of passively accepting fate out of necessity to conform to natural trends, there is actually the latent notion that fate can be changed through the process of cultivation. In other words, one can understand the mission bestowed upon oneself as a person by heaven. As Mengzi stated, “To exhaust one's mind to know one's nature is to know Heaven.^3^” When faced with troubles, people should be able to self-reflect, and consider whether they have carried out their obligations, and whether they have been diligent in the existing constraints of destiny. This is the practice of using knowledge of fate to inspire one's active bearing of responsibilities. In doing so, when there are problems in interpersonal interaction, one would be able to convert grudges to gratitude, and repay the other with a grateful heart; this would be another elevation of the self. However, this assumption of responsibility is not easy, so it is unnecessary for people to be excessively insistent. At this point, one may experience a peaceful state of mind under the principle of “man proposes, Heaven disposes.” When necessary, people should let go and let nature run its course. Here, the Confucian thought of “exhaust one's abilities” is connected to Buddhist and Daoist thought, as people learn to not insist on action, because ultimately all reality is transient.

Therefore, both the action and inaction of people faced with problems may have a positive psychological significance in Chinese cultural societies. This paper postulates that the main means of cultivation for people in Chinese cultural societies to put them at ease, is to first access the nature-abiding character of Daoism: learning to see things without expectations. Then, introduce Buddhist thought to analyze the causes and consequences in the problems, which would enable them to accommodate the other person's position. If they believe that they have not done enough, they should take more active action to fulfill their responsibilities. This is the application of Confucianism. Finally, in terms of overall reflections, they can learn to not overemphasize any specific relationship. From this, they are able to achieve the benefits of “self-cultivation” in psychological adjustment faced with interpersonal problems, and are able to express the wisdom unique to Chinese cultural tradition.

Based on the description above, we constructed a tentative theory to illustrate how *yuanfen* which comprises elements of all the above-mentioned schools of thought can draw on in specific situations to take the most suitable coping actions in Chinese cultural societies.

## Explanation of model content

In Figure 1, the model stresses that there are primarily four stages in the process of psychological adjustment when dealing with interpersonal problems in Chinese cultural societies: (1) Occurrence of an external event; (2) The cognitive processes of the culturally-inclusive wisdom; (3) The archetype of ideal responses after self-integration; (4) Achievement of psychological ease. It postulates that when people who live in Chinese cultural societies encounter relational problems, they tend to first access the cultural schema of *yuanfen*, in which *yuan* is originally a Buddhist thought, believing that everything has causes and consequences. The internal mechanism is the view of the will of Heaven though acceptance of fate (*ren-ming*) (Hsu, 2012). This is practiced through passively bearing (Hwang, 1978), being concerned with the other or related others. As a result, people are willing to undergo mental and physical training through experience to express one's mercy. Bearing bitterness transforms one's thoughts, and implies that all difficulties can be alleviated with time. This type of passive prosocial coping strategy (Chen, 2004) can help one to take some ideal coping forms to face interpersonal problems after reflection on the cultivation process. As a result, this reduces the potential for anger and fury, thereby achieving psychological adaptation. In contrast, *fen* primarily arises from the view of ethical obligation in Confucianism, emphasizing the virtue and obligation that should be fulfilled in relationship. Therefore, its internal mechanism is the heavenly will of understanding fate (*zhi-ming*) (Hsu, 2012), or practice through actively facing reality (Hwang, 1978). The subjects of consideration include not only others, but also themselves. Therefore, active exertion of the self and utmost sincerity can be used to transform thoughts and current conditions. If one can bravely shoulder responsibilities and obligations, one would take ideal coping strategies, in turn reducing the potential for regret and complaints and achieving psychological adaptation.

## Ideal coping actions after self-integration

Seeking “harmony” is a positive and auspicious social value in Eastern Asian culture. It is also a common mindset among interpersonal relationships (Huang, 1996). This model infers that, in Chinese cultural societies where relationship doctrine and interpersonal harmony are advocated, when people are dealing with relationship problems, it is common to respond with the concept of harmony achieved by negotiation. In other words, people emphasize on using courtesy but no conflict to establish harmonious interpersonal relationships and social order. The ideal interpersonal responses of “tolerance” and “forgiveness” are therefore especially respected. Since harmony is a dynamic experience, not a static structure, in Chinese cultural societies (Huang, 1996), people emphasize on the need to actively return a favor, in order to properly fulfill the responsibilities and obligations in relationships. As a result, “making effort” and “having gratitude” are ideal interpersonal responses. Based on the above, the measurement for “ideal coping actions” in this model mainly aim to include “tolerance,” “forgiveness,” “making effort,” and “having gratitude” as the primary moral acts. This model holds that these actions can be inspired through the belief in *yuanfen*, and in turn help people face interpersonal problems and maintain harmony. Their individual definitions and connotations are described as follows:

### Tolerance

The term “tolerance” for most people can be easily associated with a sense of depression, grievance, and pain. In fact, to tolerate something, one must go through four psychological mechanisms, namely “restraint,” “determination,” “acceptance,” and “retreat,” in order to allow this person to control a particular psychological intention out of this person's own will (Lee and Yang, 1998). Besides, “tolerance” has been highly praised to be an important value and philosophy in Chinese cultural societies. If people can observe the limitations of their situations in reality, then people can learn how to be indisputable and conform when they are faced with difficulties, even if they have to deal with humiliation. However, in Chapter 73 of *Daodejing*, it says, “The heavenly way is to not compete but be good at winning, not speak but be good at responding.” In addition, in “*Man in the World, Associated with Other Me*” by Zhuangzi:, “Know that there is no alternative to people acting as they do, and rest in it as what is appointed; this is the highest achievement of virtue.^4^” These mean that “tolerance” can help individuals to reach beyond their spiritual level, and highlight their integrity and cultivation in dealing with situations. In this instance, “tolerance” elevates virtue, and it is no longer merely about sacrifice or humiliation. It is valuable for individual achievement and happiness, and it also has the benefit of maintaining relationships and gaining praises from society(Lee and Yang, 1998). It can be regarded as one of the “ideal coping actions” when people are faced with challenges in relationships.

### Forgiveness

“Forgiveness” is a universal value. However, in Chinese cultural societies, the way that people express love can be very unique. Although, Confucianism emphasize on “the principle of loyalty and benevolence,” Confucius also stated that, “Only the benevolent is capable of truly loving or hating a person,^5^” which means that only a person who truly is benevolent, virtuous, and well-cultivated can be fair and selfless to like or dislike others in the correct way, and can then be close to the good ones and stay away from the malevolent ones. He also emphasized: “If a man sets his heart on benevolence, he will not do evil.^6^” This means that if a person is determined to pursue the path for benevolence, then this person will not have any dislikes toward anyone. Further, this person can help the malevolent to transform and become benevolent. In Buddhist teachings, there is a saying that “a butcher becomes a Buddha the moment he drops his cleaver.” This means that everyone has a chance to attain enlightenment and become a Buddha through cultivation. As long as people are willing to repent, then they can change their path to the “Bodhisattva path.^7^” However, they still need good cause to make this good consequence happen. To “forgive” someone can be just simple as not giving this person a hard time, but it does not mean complete “absolution.” In Chinese Societies, people can use “forgiveness” to save the “face” of the other party, and use that to maintain the harmony and ensure space for future development of the relationship.

### Making effort

The meaning of “making effort” contains the Confucian concepts of “moral and ethical obligation” and “exertion of the self” (Hwang, 2012). “Moral and ethical obligation” encourages people who live in Chinese cultural societies to have the habit of regarding “making effort” as an important virtue and obligation when dealing with people and handling situations. “Exertion of the self” focuses on the fact that individuals must mindfully obtain a profound understanding of their own human nature, so that they can understand the meaning of life. Influenced by Confucian concepts, while people who live in Chinese cultural societies obey the mandate of Heaven, they often make effort to respond to challenges. After people have fulfilled the responsibilities of their roles, then they are worthy of others. Through, the process of exerting oneself and making the best effort, they can then be worthy of themselves and have no regrets.

In addition, researchers Luo et al. (2010) used the “Hope Theory” of Snyder (2000) and proposed that the hopes in the Chinese society should contain two factors, namely “beyond adaptation” and “relentless effort.” Different to western society where “making effort” is regarded as one's internal characteristic, people regard “making effort” as a method to improve one's ability in Chinese society. It is also an attitude of noble spirit. People who silently make effort firmly and persistently, overcome challenges, and work toward their goals are worthy of praise. In other words, people who can continue to make effort when they are in a challenging situation are regarded as self-cultivated gentlemen with virtues. Since hope is most commonly raised during difficult situations, “making effort” is bound to become a method for adjustment when facing challenges in relationships.

### Having gratitude

The concept of “gratitude” is commonly discussed in Buddhist way of thinking, which emphasizes that people should have gratitude to their parents, teachers, nation, and all the sentential beings. In addition, “having gratitude” is associated with the concept of “repaying.” A person is regarded to be virtuous only if they understand the need to repay the favor when others have provided benefit to them. “Having gratitude” is not only an important guideline for interpersonal interactions, but it is also important for the self-cultivation of virtues when interacting with others and handling situations (Wood et al., 2007). In addition, it promotes harmony in relationships. Under the influence of Confucianism, if a person can maintain a kind heart, and in a relationship this person is actively aware of the responsibilities of the role they play, then this person can “restrain self and return to the rites,^8^” and have filial piety toward parents, be friendly to brothers and sisters, be trustworthy to friends, be loyal to the nation, and be kind to others. As a result, if people are actively aware of the benefits that they have received from others in the past, and due to their inner sense of sincere, or the favor they owe, they can encourage themselves to have a sense of gratitude and act accordingly.

Based on the “Resource theory” for social exchange (Foa and Foa, 1980), in addition to specific exchanges that are more about concrete resources, for example, status, money, etc., there are also a considerable amount of emotional exchanges that are based on love and affection. We suggest that “having gratitude” is also a form of emotional exchange during the process of interaction in relationships. Through this kind of interaction, it can help people to retain the initiative to take responsibilities, even if they are frustrated in their relationships—especially expressive tie with parents, spouse, partner, best friends, etc. People can transform their negative emotions into the ideal coping actions if they have gratitude when they are faced with relationship issues.

## The effect of dialectical thinking of belief in *yuanfen* in psychological adaptation

Previous studies indicated that the belief in *yuanfen* is related with psychological indicators in mental health, work satisfaction, and well-being (Lee and Chen, 2006, 2009). We postulate that *yuanfen* is correlated with psychological health is because of its latent character of dialectical thinking.

Sociologist Lee (1995) proposed a view of “fatalistic voluntarism,” and argued that Chinese beliefs in fate contain both passive fatalism and active voluntarism, which implies a kind of dialectical thinking. Dialectical thinking has shown a positive correlation with coping flexibility (Cheng, 2003, 2009); in turn, coping flexibility is an effective indicator of psychological adaptation. Therefore, we anticipate that *yuanfen* may facilitate coping flexibility, and can be used to adjust psychological stress when one is faced with relational interaction problems.

Dialectical thinking refers to the cognitive style for acceptance of opposing positions in all things. Because the world is always changing, many seemingly contradictory or opposing views are actually mutually beneficial and reliant, each with inherent positive values (Peng and Nisbett, 1999). Although, these oppositions or contradictions are classified as good or bad, primary or subordinate, or positive or negative, their natures may change after a certain degree of evolution. Through the organic process of mutual infiltration and transformation, a higher form is achieved, thereby resolving the dilemmas in a changing world (Huang, 2006).

Coping flexibility can be described as an individual's ability to take different coping strategies in different contexts; it has been discovered to be clinically related to mental and physical health (Cheng, 2001; Vriezekolk et al., 2012). Most Western literature divides coping strategies into “active problem-focused coping” and “passive emotion-focused coping,” and claims that the former produces more ideal psychological adaptation (Lazarus and Folkman, 1984; Folkman and Moskowitz, 2004), though there is no stable consensus on this view (Carver et al., 1989). Empirical studies showed that “active problem-focused coping” actually has a low positive correlation with psychological adaptation among children or adolescents with less social experience (Clarke, 2006). This fact suggests that the oppositional perspective of dichotomy is insufficient to explain the functioning of coping flexibility, and the factors of social context cannot be overlooked. In other words, “active” is not necessary positive, and “passive” is not necessary negative, the vision of dialectical thinking is broader and can better describe to the customary Chinese ways of dealing with people and events.

In Western literature, there has long been scholars that attempted to incorporate a dual-axis model of coping: the “active axis” and the “social axis” to explain the four possible types of coping strategies for interpersonal relationship (Hobfoll et al., 1994). Chen (2004) used this model as a basis to name the four coping styles: “active prosocial,” “active antisocial,” “passive prosocial,” and “passive antisocial” in Taiwan, finding that “active prosocial,” and “passive prosocial” are the greatest predictors of positive psychological health and reducers of depression and anxiety. This suggests that effective coping must account for social relationships, even though they might be passive actions such as forbearance, accommodation, and submission, all these can benefit psychological adaptation (Hsu et al., 2008).

Following dual-axis model of coping proposed by Hobfoll et al. (1994) and Hsu et al. (2008), our model in Figure 1 also makes the distinction between “active vs. passive.” Nevertheless, our model incorporated the concepts of Piaget (1977), creating the two forms of “active/accommodation vs. passive/assimilation.” The former means actively changing existing cognitive schemas to incorporate new information from the external environment, while the latter uses existing cognitive schemas to receive external information passively. Both are necessary for psychological adaptation.

Furthermore, because Chinese society is affected by Confucianism, our model postulates that the indicator of “prosocialness” would have a more complex meaning on the active coping dimension, and actions tend to be different based on the closeness of relationships. In other words, people who live in Confucian societies follow a relationalism of differentiated order (Fei, 1948; Hwang, 2012), and would do their best to maintain psychosocial homeostasis with one's own interpersonal network. Therefore, our model theorizes that the practice of “exert oneself” in Confucianism may inspire the internal to external active/accommodation adaptation in a specific interpersonal situation, because there is a sense of obligation toward the relationship in that context. Affected by Buddhist and Daoist thought, we postulate that people who live in Chinese cultural societies may tend to adopt merciful and non-confrontational strategies, while trusting in the causes and consequences of natural cycles. That is, they may prefer inaction, holding to stillness, and avoid thoughtless action, and they are able to promote the external to internal passive/assimilation adaptation path. Based on our framework in Figure 1, we conducted an empirical study to demonstrate this tentative theory.

### Empirical study based on the framework

The objective of this study was to preliminarily explore the relationship between beliefs in *yuanfen* and the ideal coping actions, on the basis of the framework. The beliefs in *yuanfen* are divided into two main types, namely the type of “*ren-ming* based on obeying fate” and the type of “*zhi-ming* based on understanding fate.” Four dimensions for measuring the ideal coping actions included: tolerance, forgiveness, making effort, and having gratitude. A scenario about family dilemma is used as an event. In this study, it is assumed that a canonical correlation exists between the beliefs in *yuanfen* and the ideal coping actions, and they are positively correlated.

## Methods

### Participants

We created the questionnaires online and published the survey link on websites, social media, and bulletin board system. There were 188 adult volunteers with 67 males and 121 females in Taiwan to complete the questionnaires. The personal information in the questionnaires covered four age groups (20–24, 25–29, 30–35, 36 and over). There were 121, 37, 28, and 2 participants in each range, respectively. As for the education, there were 62 undergraduate students, 21 participants currently enrolled in graduate schools for master degree, 3 participants currently enrolled in PhD programs, 3 participants graduated from vocational high schools, 83 participants graduated from colleges, and 16 participants graduated with master's degree. This study received Institutional Review Board (IRB) approval and participants gave informed consent.

### Materials

#### *Yuan* belief scale (Appendix 1 in supplementary material)

The items were modified based on the initial scale by Lee and Chen (2006). After the process of experts review, item analysis and factor analysis, the scale finally includes two sub-scales for “*Ren-ming* type (obeying fate)” and “*Zhi-ming* type (understanding fate).” There were a total of six questions. 1 point represents “totally disagree” and 5 point represents “totally agree,” which represents two ends of the extremes. Participants were asked to circle 1–5 based on how much they agreed with the description of the questions. We previously performed an exploratory factor analysis (EFA) on 118 adult participants. Using principle axis analysis and direct oblique rotation, two factors were extracted. The “*Ren-ming* type” subscale consisted of 3 items (α = 0.88), and the “*Zhi-ming* type” subscale consisted of 3 items (α = 0.83).

#### The scenario task and the ideal coping action scale (Appendix 2 in supplementary material)

Through the scenario, participants were asked to read the story, and imagine themselves as the main character. The details of the scenario are as follows:

It was love at first sight for you and your partner, and you have been together for many years. Recently you have plans to get married. When you excitedly try to share your happiness with your family, your mother does not like your partner, and she is also worried that you are rushing into your decision. You then started to have frequent disagreements with your mother, and your family relationship is now facing challenges. One day, after a serious and intense fight, your mother says to you suddenly, “I have done my responsibilities for raising you, but I am very disappointed in you. If you persist and want to marry your partner, then you are no longer welcomed in this family.”

We explored the meaning of the four actions in Chinese cultural societies through literature review. The content of the questionnaire were composed together with the scenario. The preliminary questions were consulted with three experts. They helped to modify and test the questions. Eventually, three questions were left for measuring each type of actions. For the assessment, 1 represents “extremely unlikely” and 5 represents “extremely likely.” Participants were asked to imagine the situation was happening to themselves, and were asked to assess the extent they would take the four actions by selecting 1–5 based on how much they agreed with the questions. The measuring questions of four actions are as follows:

1. Tolerance
  The meaning of tolerance mainly contains restraint, persistence, endurance, and retreat, which are the four psychological mechanisms for individuals to self-control their specific psychological desire(Lee and Yang, 1998). This section consisted of 3 items (α = 0.76); the average response was 10.51 (*SD* = 2.43).
2. Forgiveness
  The meaning of forgiveness mainly contains the Confucian concept of “benevolence” and the Buddhist concept of “compassion.” This section consisted of 3 items (α = 0.81); the average response was 9.86 (*SD* = 2.57).
3. Making effort
  The meaning of making effort mainly contains the Confucian concept of “moral and ethical obligation” and “exertion of the self.” This section consisted of 3 items (α = 0.76); the average response was 11.13 (*SD* = 2.61).
4. Having gratitude
  The meaning of having gratitude mainly focuses on the affection exchange in an interactive relationship, and thereby promotes harmonious relationships and transforms negative emotions. This is important in the self-cultivation of virtues. This section consisted of 3 items (α = 0.76); the average response was 11.54 (*SD* = 2.49).

### Research procedure and data analysis

The participants were first asked to read the informed consent online. After they were aware of the objectives of the study and their rights, they were guided to another link to read the scenario. After reading the task, they needed to complete the ideal coping action scale, *Yuan* belief scale, and personal details. After data collection, the statistical program SPSS 20.0 was used to process the data. Canonical correlation analysis (CCA) was used to explore the two aspects of beliefs in *yuanfen*, namely the type of “*Ren-ming* (obeying fate)” and the type of “*Zhi-ming* (understanding fate),” as well as to explore the four aspects of “ideal coping actions” as criterion variables, namely “tolerance,” “forgiveness,” “making effort,” and “having gratitude.”

## Results

Canonical correlation analysis was performed by extracting two sets of canonical variables with statistical significance (*Wilks L*. = 0.76, *p* < 0.001; *Wilks L*. = 0.94, *p* < 0.05), indicating that, for the participants, “beliefs in *yuanfen*” and “ideal coping actions” have two sets of canonical variables with canonical correlations influencing each other.

**Figure 2:**
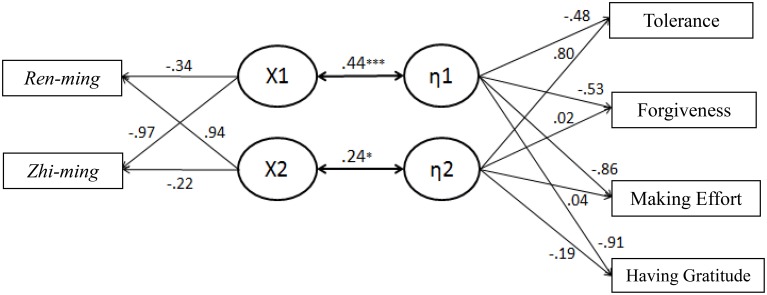
**Graphical representation of the significant canonical functions and the contributing predictors (left side) and criterion variables (right side)**. ^*^p < 0.05, ^***^p < 0.001.

From the statistical results in Table 1 and the canonical correlation structure path diagram shown in Figure 2, the canonical correlation coefficient ρ was 0.44 (*p* < 0.001) and the coefficient of determination ρ2 was 0.19, indicating that the first canonical variable χ1 of the predictor variable could explain 19% of the total variance of the first canonical variable η1 of the criterion variable, and η1 could explain 52.47% of the total variance of the criterion variable. Through the use of the first set of the canonical correlations (χ1, η1), the predictor variable could explain 10.07% of the total variance of the criterion variable, and χ1 could explain 53.18% of the total variance of the predictor variable. Through the use of the first set of canonical correlation (χ1, η1), criterion variable could explain 10.20% of the total variance of the predictor variable. The canonical correlation coefficient ρ for the second set was 0.24 (*p* < 0.05) and the coefficient of determination ρ2 was 0.06, indicating that the second canonical variable χ2 of the predictor variable could explain 6% of the total variance of the second canonical variable η2 of the criterion variable, and η2 could explain 16.85% of the total variance of the criterion variable. Through the use of the second set of the canonical correlation (χ2, η2), the predictor variable could explain 0.98% of the total variance of the criterion variable, and χ2 could explain 46.82% of the total variance of the predictor variable. Through the use of the second set of canonical correlation (χ2, η2), the criterion variable could explain 2.72% of the total variance of the predictor variable.

**Table 1:** **Canonical correlations between the belief in *yuanfen* and the ideal coping actions**.

**Belief in *yuanfen***	***n* = 188**				
	**Canonical factor**		**Ideal coping actions**	**Canonical factor**	
	***χ*_1_**	***χ*_2_**		***η*_1_**	***η*_2_**
*Renming*	−0.34	0.94	Tolerance	−0.48	0.80
*Zhiming*	−0.97	−0.22	Forgiveness	−0.53	0.02
			Making efforts	−0.86	0.04
			Having gratitude	−0.91	−0.19
Variance extracted %	53.18	46.82	Variance extracted %	52.47	16.85
Measure of redundancy	10.20	2.72	Measure of redundancy	10.07	0.98
				ρ^2^ 0.19	0.06
				ρ 0.44^***^	0.24^*^

* *p < 0.05*,

*** *p < 0.001*.

In addition, in practice, the “canonical structure coefficient,” that is, after taking the absolute value of the canonical structure loading, the variable with a larger value and a more significant meaning would be used as the reference for the naming convention for the canonical variables. From Figure 2, it can be observed that, in terms of the predictor variable, “*zhi-ming* type” had the highest correlation with the first canonical variable χ1 with a loading of −0.97, and therefore the first canonical variable χ1 was named as the “concept of internal control (voluntarism).” In terms of criterion variables, “making effort” and “having gratitude” had the highest correlation with the first canonical variable η1 with loadings of −0.86 and −0.91, and therefore the first canonical variable was named as “fulfilling one's own duties.” For this study, from the first set of canonical correlation (χ1, η1), it can be observed that, the “concept of internal control (voluntarism)” and “fulfilling one's own duties” have a close correlation. In addition, the canonical loading for the predictor variable and criterion variable both showed the same direction, indicating that they were positively correlated, which means that the stronger the “*zhi-ming* type,” the easier it is for people to “make effort” and “have gratitude.” In terms of the predictor variable, “*ren-ming* type” had the highest correlation with the second canonical variable χ2 with a loading of 0.94, and therefore the second canonical variable χ2 was named as the “concept of external control (fatalism).” In terms of the criterion variables, “tolerance” had the highest correlation with the second canonical variable η2 with a loading of 0.80, and therefore the second canonical variable was named as “the embodied way of bearing.” From the second set of canonical correlation (χ2, η2), it can be observed that, the “concept of external control (fatalism)” and “the embodied way of bearing” have a close correlation. In addition, the canonical loading for the predictor variable and the criterion variable both showed the same direction, indicating that they were positively correlated, which means that the stronger the “*ren-ming* type,” the easier it is for people to have “tolerance” as their response.

## Discussion

According to the results, when participants were facing the family relationships dilemma, “beliefs in *yuanfen*” and “ideal coping actions” had significant canonical correlations, indicating that the theoretical hypotheses in this study have obtained preliminary empirical validation. The “*ren-ming* type” (obeying fate) may support actions that are related to “the embodied way of bearing,” and “*zhi-ming* type” (understanding fate) can initiate actions that are related to “fulfilling one's own duties.”

This study has found that, in regards to “beliefs in *yuanfen*” and “ideal coping actions,” two sets of canonical variables could be successfully extracted to achieve statistical significance. Based on the canonical loading, when people face family dilemma, those with a stronger “*zhi-ming* type,” the easiest coping initiated is “having gratitude,” followed by “making effort.” It confirms that when facing their family dilemma, “having gratitude,” and “making effort” can be initiated by one's “internal control (voluntarism).”

The loadings for “forgiveness” and “tolerance” were greater than 0.40, indicating that there was also a positive relationship with “*zhi-ming* type.” Although, “forgiveness” and “tolerance” seemed as the result of “the embodied way of bearing,” in practice, when participants face their family and love relationship dilemma, they are more likely to cope the situation by the strategy of “internal control (voluntarism),” in which “tolerance” has both the characteristics of “passively bearing it” and “actively taking responsibilities.” However, the loading for “forgiveness” and “tolerance” for the first set of canonical correlation was significantly lower than that of “having gratitude” and “making effort.” Therefore, in this study, “forgiveness” and “tolerance” were not included as part of the naming convention for the first set of canonical factors.

For the second set of canonical factors, it can be observed that, “*ren-ming* type” and “tolerance” indeed had a stronger correlation, indicating that when participants face their family and love relationship dilemma, “tolerance” originates from strong “*ren-ming*,” and therefore is more likely to be seen in people who perceive that they are “passively bearing it.” In terms of “forgiveness,” the relationship with “*ren-ming*” was not significant, but the correlation was stronger with “*zhi-ming*,” indicating that “forgiveness” can be regarded as an action that people are “actively taking responsibilities,” and must be initiated by “*zhi-ming*.”

This study is limited to family relationships. Even if the relationships between children and their parents are full of tension, under the influence of the Confucianism, despite the fact that parents make mistakes, children have a unbreakable bond with their blood-related parents, and they cannot use “forgiveness” to treat or interpret their relationship with the parents. It is obvious that for the relationship between children and parents, the self-cultivation involved for “forgiveness” is more complex, and can be explored in future studies.

Taking a further look at the results, one's “internal control (voluntarism)” and “fulfilling one's own duties” was positively related to each other. It shows that, in a family relationship, if one is “fulfilling one's own duties” in terms of “making effort” and “having gratitude,” then one must have the “internal control (voluntarism).” On the other hand, the “external control (fatalism)” and “the embodied way of bearing” were positively correlated. This means that, in a family relationship, if one can “tolerate,” then one must have the “*ren-ming*.” Conceiving in terms of Mandala model of self (Hwang, 2011), it indeed shows that the “cultural wisdom” with regards to “beliefs in *yuanfen*” can be a key strategy for “ideal coping actions” upheld by Chinese cultural societies. At the same time, when placing the research results in western consultative theories, it confirms that, cognitive beliefs triggered by external events is often related to the consequences of subsequent actions, as well as how people adapt psychologically. Such influence is more significant in major events relating to close relationships (Beck, 1989). As a result, people's cognitive interpretation of events is indeed the key for moving toward psychological homeostasis. In our study, “beliefs in *yuanfen*” was an expression for the state of awareness, which included “*ren-ming* type based on obeying fate” and “*zhi-ming* type based on understanding fate.”

In addition, this study identified that when families experience challenges, “*zhi-ming*” promote the initiation of “forgiveness.” In other words, the meaning of “forgiveness” is more about the “concept of internal control (voluntarism),” which can be considered to be actively “fulfilling one's own duties,” rather than passively “the embodied way of bearing.” From the common sense perspective, “forgiveness” indeed has the meaning of “bearing with the embodied way.” However, under the influence of Confucianism on “benevolence” and “the differential mode of association” in Chinese society (Fei, 1948; Hwang, 2012), it is not agreed that everyone is entitled to be “forgiven,” or that every situation is worthwhile or acceptable to discuss “forgiveness.” Just as stated in *The Analects* that people should recompense injury with justice, and recompense kindness with kindness which explains that people should use “justice” but not “forgive” in response to those who have harmed them. If people easily forgive those who have harmed them, then although it may appear as if it is an act of great kindness, it can inevitably cause confusion^9^. Seen in this light, “forgiveness” is not purely a mediating method for “passively bearing it.” Instead, it requires a long period of time with deep contemplation in order to “actively take responsibilities” for one's action. In other words, “forgiveness” is not just tolerating with a sense of helplessness, but instead, people can choose to forgive. This means that people recognize that in terms of their responsibilities for the relationship, they are adopting the “fulfilling one's own duties” approach. As a result, this study revealed that “forgiveness” had a higher correlation with “*zhi-ming* type.” In other words, people can have a higher order of self-awareness and a better integration with the concept of “fate.” It is recommended that future studies can further explore the meaning of the active adoption of “forgiveness” in various relationships. In terms of the passive adoption of “forgiveness,” it is recommended to use “avoidance” as the subsequent measurement. This is due to the fact that, for the interactions within relationships in Chinese cultural societies, people's objective is pursuing harmony and avoiding conflicts (Huang, 1996). As a result, it is reasonable to define “avoidance” as the passive approach. However, it requires in-depth literature exploration and researches to clarify that avoidance is the optimal ideal coping action.

When conducting this study, economic considerations and timeliness must be taken into consideration. Future studies should use a more optimal sampling method to improve the ecological validity. In addition, one should consider different age groups and the balance between genders, so that the sample will be more representative. A longitudinal study that involves repeated observations of the same variables may help to know if the tendencies would change over time. If one wants to expand the results to societies with Chinese cultures, then samples from the following areas should be collected: China, Hong Kong, Macau, Singapore, etc. If adequate data can be collected, then it will provide more meaningful results, and one can compare the difference between regions.

Secondly, this study mainly focused on validating the theoretical model used in previous literature. Therefore, for the empirical exploration, the variables were limited to the relationship between two beliefs in *yuanfen* and four ideal coping actions. However, in practice, it can be other important influencing factors, for example, personality. Since Eastern indigenous psychology usually focused on the importance of the situation, it may cause “situational bias,” and ignore the fact that personality can have a mediating effect for initiating actions. It is recommended that in future studies, personality can be included as one of the measurement variable.

Finally, this study suggests that for any constitutional research on psychology focusing on cultural wisdom, regardless of the objectives or methods, it is recommended that each researcher should have an appropriate respect. In other words, whether the cultural wisdom has any meanings for its existence in the world people live in, it also contains an element called “insight” which is difficult to measure. Taking this study as an example, only when people realized karmic affiliation can they then know how to stop in the utmost excellence (*Zhizhi*), and developing a different mindset or behaviors through the process of “meditation, peacefulness, calmness, contemplation, acquisition,^10^” in order to effectively adapt psychologically. In other words, people cannot obtain actual benefit in real life by just understanding karmic affiliation literally. Instead, to a certain extent they need to go through the process of “self-cultivation.”

*Zhizhi* can have two interpretations. One means that people can use the concept as the objective of “self-cultivation,” and this is the most important agenda proposed in *Great Learning*: “The way of great learning consists in manifesting one's bright virtue, consists in loving people, and consists in knowing to rest in the utmost excellence.^11^” At the same time, it also stresses on the importance of implementing appropriate interactions when dealing with people and situations, as well as taking responsibilities of the relationship (Fu, 2013). The second meaning can be explained in general terms, “knowing when is the appropriate time to stop,” “knowing the limitations of the boundaries,” “knowing how much you can advance and how much you can retreat.” As chapter 44 in *Laozi* stated, “Being contented with one's lot, one will not be disgraced by others for it. He who is content with his lot will not be humiliated. To be always contented means a lifetime without disgrace.” In other words, people should know how to be less selfish and have fewer desires in order to stay away from danger, which also include the fact that people should exercise their restraint to “lust,” just as the concept of the Buddhist study on “*The twelve links of cyclic existence*”: “you can be liberated if you do not grasp on attachment and you can let it go, in other words, when you have desire but you do not take it as an attachment, then there is a way of eliminating it” (Bhikkhuni et al., 2008). In other words, once you attain insight of the karmic phenomenon of creation and elimination for various complex relationships, the path to liberation and open mind can then be created. As a result, in general, any research can only present part of what is actually there from a particular perspective, which means that it can only show the knowledge that the researchers are trying to establish themselves. If researchers can accomplish self-realization through their research activities, and try to have the knowledge that they have established to be used by people in practice, then they can promote the research results from a scientific micro world into practical activities in real life. Therefore, there will be opportunities for scientific micro worlds with various fields and perspectives to interact with each other, in order to achieve the objectives of implementation (Shen, 2002). This is the concept of knowledge upheld in this study. It is recommended that future research can follow the concept of “one mind, many mentalities” (Shweder et al., 1998). Starting from the social environment that one is currently in, and the search for the research topic that is in line with the mindset generally accepted by people, while explaining the unique cultural mentality in local societies. Based on this, one can establish unique theories or perspectives, and perform empirical research. At the same time, one can pay attention to the source of samples and sample characteristics, as well as various limitations for derivations, in order to propose a research finding that is more innovative and unique, in the development of worldwide knowledge of psychology.

## Conclusion

On the premise of fate as part of life, *yuanfen* comprises the various life experiences of people who live in Chinese cultural societies that connect various tangible and intangible matters and drive good and bad relationships. Therefore, there are qualitative differences in the nature of *yuanfen*. Negative *yuanfen* is likely to bring people pain, and should be cut off rationally. However, Chinese cultural societies places emphasis on virtue, and is affected by the Buddhist view of cause and consequences and the Daoist view of following nature. Thus, unlike the decisiveness with which interpersonal problems are handled in the West, it is usually difficult for people in Chinese cultural societies to sever a relationship that is not good enough, rather opting to maintain the relationship after seeing it as a necessary trial in life. The various psychological and cultural factors involved still need to be clarified.

In addition, *yuanfen* is not an absolutely unreachable concept in Chinese cultural societies. People usually believe that those with “beneficial *yuan*” have the opportunity to change their fate (Wang, 1987), so it can be used to explain various types of relational interactions, and can be a life attitude (Chang and Holt, 1991). However, further exploration is required to assess whether knowledge cumulating from cultural traditions as well as the wisdom derived therefrom, both have positive effects on psychological health or whether they can produce negative effects under certain circumstances. This is necessary in order to propose relational theories or psychological treatment regimens with greater cultural compatibility.

Based on the cultural view, this article proposes a dialectical model to interpret the construct of *yuanfen* and the psychological adaptation processes in interpersonal interaction in Chinese cultural societies, and it also provides a tentative framework for understanding people in non-Western societies. In short, we define *yuanfen* as serendipity in relationship, an important cultural wisdom, flexible determinism and a way of psychological adjustment when dealing with interpersonal issues in Chinese cultural societies. In today's global communities, indigenous and cultural perspective toward social and personal relationship is indispensable. This framework may contribute to the development of relational studies, but in need of more empirical verification.

## Author contributions

All authors listed, have made substantial, direct and intellectual contribution to the work, and approved it for publication.

### Conflict of interest statement

The authors declare that the research was conducted in the absence of any commercial or financial relationships that could be construed as a potential conflict of interest.

## Footnotes

^1^References to this passage is based on *Liji Daxue pian* of Confucianism, as quoted in Cua (2003, p. 47).

^2^This aphorism can be found in the *Chinese Buddhist Canon (Dazhengzang)*, 47, 259. [ ， 47 ， 259 ◦].

^3^See *Mengzi*, Jin Xin I, chap 1, as quoted in Sturgeon (2011).

^4^See Chapter 73 of *Daodejing* and the Inner Chapters of *Zhuangzi* of Daoism, as quoted in Sturgeon (2011).

^5^See *The Analects*, Li Ren, chap 3, as quoted in Sturgeon (2011).

^6^See *The Analects*, Li Ren, chap 4, as quoted in Sturgeon (2011).

^7^There are the six perfections in practice to complete Bodhisattva path: generosity, morality, patience, energy, meditation, and wisdom, as quoted in Trungpa (2013).

^8^See *The Analects*, Yan Yuan, chap 1, as quoted in Sturgeon (2011).

^9^See *The Analects*, Xian Wen, chap 34, as quoted in Sturgeon (2011).

^10^References to this passage are based on *Liji Daxue pian* of Confucianism, as quoted in Cua (2003).

^11^See *Liji*, Daxue, chap 1, as quoted in Sturgeon (2011).
