# Supplementary material for: Serendipity in Relationship: A Tentative Theory of the Cognitive Process of Yuanfen and Its Psychological Constructs in Chinese Cultural Societies
Source: Front Psychol. 2016 Mar 1;7:282. doi: 10.3389/fpsyg.2016.00282 (PMC4771764; doi:10.3389/fpsyg.2016.00282)
Supplement: Supplementary file 1 [file DataSheet1.docx]

**Appendix 1**

**Yuan Belief Scale**

◎How to answer the questions:

The following questions are to understand your perspective. Please read each line carefully, and based on how much you agree with each of item, please circle your selection. Please do not consider other people’s perspectives, and only answer based on your own perspective.

**Totally disagree Totally agree**

| 1. For all relationships, getting together and getting separated are all due to karmic retributions, so even if it is a dilemma, you just need to accept it. | 1 | 2 | 3 | 4 | 5 |
| --- | --- | --- | --- | --- | --- |
| 1. All the failures and achievements in this life are the results of your karma from previous lives, and people are forced to accept the current situation. | 1 | 2 | 3 | 4 | 5 |
| 1. All the love and hate relationships in life are predetermined by fate, and you cannot change them. | 1 | 2 | 3 | 4 | 5 |
| 1. Friends are gifts from the heaven. Actively networking is the way to obtain abundant human resources. | 1 | 2 | 3 | 4 | 5 |
| 1. For every relationship, even when there are unavoidable problems, as long as you work hard, you can improve it. | 1 | 2 | 3 | 4 | 5 |
| 1. If you work hard, then you will able to reverse the fate that was predetermined in any relationship established. | 1 | 2 | 3 | 4 | 5 |

**Appendix 2**

**Ideal Coping Actions Scale**

◎How to answer the questions:

You will read a short paragraph below. Imagine you are the main character in the story. After you finish reading, please answer the following questions.

*It was love at first sight for you and your partner, and you have been together for many years. Recently you have plans to get married. When you excitedly try to share your happiness with your family, your mother does not like your partner, and she is also worried that you are rushing into your decision. You then started to have frequent disagreements with your mother, and your family relationship is now facing challenges. One day, after a serious and intense fight, you mother says to you suddenly, ‘I have done my responsibilities for raising you, but I am very disappointed in you. If you persist and want to marry your partner, then you are no longer welcomed in this family.’*

Imagine if the event mentioned above is happening to you, what are the probabilities for you to take the following responses in order to adapt your mindset?

**Extremely unlikely Extremely likely**

| 1. In order to maintain harmonious relations, you are willing to choose to tolerate. | 1 | 2 | 3 | 4 | 5 |
| --- | --- | --- | --- | --- | --- |
| 1. Don't fight with your mother, because taking a step backward opens up a wider world. | 1 | 2 | 3 | 4 | 5 |
| 1. Try to restrain negative emotions and behaviors as much as possible. | 1 | 2 | 3 | 4 | 5 |
| 1. Although you have the feeling of being hurt, you are willing to forgive your mother. | 1 | 2 | 3 | 4 | 5 |
| 1. Use your empathy to understand and accept your mothers' behavior. | 1 | 2 | 3 | 4 | 5 |
| 1. With the heart to obey, you respect any decision made by your mother. | 1 | 2 | 3 | 4 | 5 |
| 1. You believe that there must be a way to overturn the tension in the relationship with your mother. | 1 | 2 | 3 | 4 | 5 |
| 1. You feel obligated to take up the responsibilities in changing the relationship with your mother. | 1 | 2 | 3 | 4 | 5 |
| 1. Actively taking various approaches and try your best to improve the relationship with your mother. | 1 | 2 | 3 | 4 | 5 |
| 1. You are able to regard the challenges in family relationship as a positive life experience. | 1 | 2 | 3 | 4 | 5 |
| 1. Even if the relationship changes, you still feel grateful for your mother’s efforts in the past. | 1 | 2 | 3 | 4 | 5 |
| 1. You are more appreciative in regards to family relationship and you want to express your good faith by returning favors. | 1 | 2 | 3 | 4 | 5 |
